# Sialylated Clusterin Binds CD33 to Regulate Microglial Functions in Alzheimer's Disease

**DOI:** 10.1002/alz70855_100929

**Published:** 2025-12-23

**Authors:** Kanayo Satoh, Ye Zhou, Roger B. Dodd, Masahiro Enomoto, Yalun Zhang, Christopher Bohm, Fusheng Chen, Seema Qamar, Mamunur Rashid, Jean Sevalle, Beatrice Acheson, Deniz Ghaffari, Jennifer K Griffin, Mark Wilson, Paul E Fraser, Elizabeth M Bradshaw, Peter St George‐Hyslop

**Affiliations:** ^1^ University of Toronto, Toronto, ON, Canada; ^2^ Cambridge University, Cambridge, Cambridge, United Kingdom; ^3^ University Health Network, Toronto, ON, Canada; ^4^ Columbia University, New York, NY, USA; ^5^ University of Wollongong, Wollongong, Australia; ^6^ Department of Neurology, Columbia University, New York, NY, USA

## Abstract

**Background:**

The sialic‐acid binding immunoglobulin‐like lectin 3 receptor (Siglec‐3 / CD33) expressed on microglia, regulates immune functions relevant to Alzheimer's disease (AD). Clusterin (CLU) and apolipoprotein E (ApoE) are soluble, sialylated proteins implicated in AD pathogenesis through genetic associations and their interactions with amyloid‐beta (Aβ). However, the role of these proteins as potential CD33 ligands remains unclear. This study explores whether CLU and/or ApoE bind CD33 and examines the functional impact of these interactions on Aβ uptake and amyloid plaque clearance.

**Methods:**

The binding of CD33 to CLU and ApoE was assessed through co‐immunoprecipitation using U937 cells (endogenously expressing CD33) and HEK293 cells (expressing exogenous CD33). Quantitative bio‐layer interferometry (BLI) and microscale thermophoresis determined binding affinities, focusing on the role of CD33's Arg119 sialic acid binding site. In situ proximity ligation assays (PLA) and co‐immunoprecipitation from AD and control human brain lysates validated in vivo interactions. Functional assays examined Aβ uptake and amyloid plaque clearance in monocytes and U937 cells, with or without CLU treatment.

**Results:**

The quantitative binding assay revealed that CLU, but not ApoE, was a sialylation‐dependent ligand for CD33, binding with high affinity (Kd = 28.9 ± 10.3 nM). Binding required an intact Arg119 residue and dimeric CD33 structure. PLA and co‐immunoprecipitation studies demonstrated colocalization of CD33 and CLU on microglia in AD brains, especially near amyloid plaques. Functionally, sialylated CLU inhibited Aβ uptake in monocytes from CD33 “CC” risk allele carriers and reduced amyloid plaque clearance in U937 cells. Desialylated CLU showed no significant effects. Notably, CLU + Aβ oligomers induced stronger CD33 ITIM signaling than CLU alone, enhancing phosphorylation and SHP‐1 recruitment.

**Conclusion:**

This study identifies CLU as a specific CD33 ligand and highlights its role in modulating microglial functions via CD33 ITIM signaling. Sialylated CLU inhibits Aβ uptake and amyloid plaque clearance, suggesting a potential mechanism underlying microglial dysfunction in AD. These findings underscore the therapeutic potential of targeting the CD33‐CLU axis to restore microglial homeostasis and enhance amyloid clearance in AD.

